# Editorial: Advances in Rowing Physiology

**DOI:** 10.3389/fphys.2022.939229

**Published:** 2022-05-26

**Authors:** Stefanos Volianitis, Yiannis Koutedakis, Niels H. Secher

**Affiliations:** ^1^ Department of Physical Education, College of Education, Qatar University, Doha, Qatar; ^2^ Department of Physical Education and Sport Science, University of Thessaly, Trikala, Greece; ^3^ Department of Anesthesiology, Rigshospitalet, Institute for Clinical Medicine, University of Copenhagen, Copenhagen, Denmark

**Keywords:** Pacing, energy cost, overtraining, central fatigue, rowing ergometer

## Introduction

Almost 100 years ago, it was considered that *“the rowing of a crew in a racing shell with sliding seats is a form of exercise in which a greater total energy expenditure is attainable, for periods of five to 20 min, than under any other conditions. No other exertion comes so near to bringing the entire muscle mass of the body into maximal extension and contraction”* (Henderson and Haggard, 1925). Since then many studies confirmed this notion and showcased rowing as *“the ultimate challenge to the human body”* (Volianitis and Secher, 2009). The articles in this Research Topic address a range of questions relevant not only to Olympic rowing performance, but also to the recently increasingly popular indoor rowing.

## Featured Publications

The usual pacing pattern of elite competitive rowers, regardless of finishing position or sex (Garland, 2005), has been to row the first 500 m at a significantly faster pace than subsequent sections of a 2000 m race (Secher et al., 1982). Although there are notable tactical and psychological reasons for starting fast, at least for on-water rowing where the leader has visual control of the competition, it is not easy to identify physiological reasons why this has been the adopted strategy, as the power relationship between energy demand and speed of the boat should favor a more even pacing. Boillet et al. evaluated physiological and psychological responses to a rowing ergometer race using different pacing strategies (i.e., the “positive-split” compared to a “negative-split” or a “constant-split”). The race distance used in the study (1,500 m) is both a limitation and a strength, as the shortened distance has been selected for the Los Angeles 2028 Olympic Games. The “positive-split” strategy is associated with high blood lactate and high exertion levels and is the least appreciated by rowers. One speculative explanation why rowers are using the seemingly more “painful” pacing pattern could be offered by the association of rowing performance with the total amount of oxygen consumed during the race. An initial spurt allows for larger total volume of oxygen consumed and power produced, compared to a more even pacing (Volianitis et al., 2020).

However, the study of Mentzoni and Losnegard that analyses the pacing patterns of rowers in A-finals of recent World and European championships reveals that medalists currently adopt a more even pacing profile compared to that of the fourth–sixth place finishers, confirming the theoretical expectation that such pacing profile would be advantageous in rowing. Furthermore, considering that such even pacing discriminates competitors in World class rowing, it also suggests that the capability to cope with the possible mental challenges (e.g., maintaining confidence when not leading) maybe a trait of successful rowing performance.

The energy expenditure of elite open-class male rowers is extraordinarily high and it is predominantly supported by carbohydrate oxidation, as estimated by Winkert et al. Thus, a single training session may potentially precipitate glycogen depletion, indicating that availability and replenishment of glycogen stores may be a key factor for successful rowing training. Additionally, and perhaps more importantly, such high energy expenditure approaches the suggested maximum alimentary sustainable energy supply (∼3 times the resting metabolic rate, Thurber et al., 2019), supporting the notion that there may be an upper energetic limit to rowing training volume (Mader and Hollmann, 1977). In this context, the study of Turner et al. suggests that inclusion of high intensity training can improve 2,000 m rowing performance (Ní Chéilleachair et al., 2017). Considering that high intensity training can reduce glycogen utilization during exercise of similar intensity (Burgomaster et al., 2006), it could provide a feasible training alternative when the energy demands of extensive low intensity training volume approach the biological alimentary ceiling.

The energy cost of rowing (ECR) has been described for on-water rowing, albeit by modeling the metabolic demand, from the estimated mechanical power required to maintain a given speed against estimated resistance, instead of measuring it (di Prampero et al., 1971). The study by Blervaque et al. is the first to evaluate the ECR for ergometer rowing, taking into account both the measured oxidative and the estimated glycolytic non-oxidative components. The findings demonstrate that ECR is negatively correlated with rowing performance but positively correlated with contribution of fat oxidation to energy supply in moderate-intensity exercises. These associations support the notion of metabolic flexibility (i.e., the ability to switch back and forth between lipid and carbohydrate oxidation, depending on energy demand and substrate availability at higher absolute workloads, Storlien et al., 2004) that has been shown across individuals of widely different metabolic capabilities (San-Millán and Brooks, 2018) and showcase its presence in elite rowers.

The power relationship between VO2 and boat velocity (Secher 1983) would predict an ergogenic effect of oxygen supplementation on rowing performance, albeit not obligatory (Volianitis et al.). Due to the synchronous movement pattern of the limbs during rowing, it seems that there is a central constraint preventing recruitment of the leg muscles during the two-legged exercise inherent to rowing (the leg “strength paradox,” Secher 1975), whilst the arm muscles are not constrained by such central activation. Considering that the largest amount of work during rowing is performed by the large leg muscles, this unique neuromuscular constrain likely explains why an increase in VO2max does not necessarily increase the amount of work performed during rowing.

Another intervention that potentially has ergogenic effect on rowing performance is bicarbonate supplementation by means of enhanced blood buffer capacity and attenuation of fatigue (Nielsen et al., 2002). The study by Nielsen et al. estimates rowing-induced changes in plasma volume, induced by the rapid fluid-shift out of the blood into the tissues during even short-term maximal exercise (Kaltreider and Meneely, 1940), and suggests that administration of sodium bicarbonate is associated with attenuated decrease in plasma volume. The implication of these estimates is that studies evaluating the effect of sodium bicarbonate on performance should account also for plasma volume changes.

Overtraining and the associated symptoms of prolonged fatigue, trainability loss, decreased levels of recovery and unexplained strength declines often appears in elite competitors (Koutedakis and Sharp, 1998), including Olympic rowers (Koutedakis et al., 1995). Bizjak et al. assessed inflammatory and immunological markers to monitor cumulated training stress in highly trained rowers during competition vs preparation (i.e., high vs low metabolic stress) phases. The authors suggested that assessment of damage-associated molecular patterns, cytokines and cell surface expression of cellular immune markers are sensitive to the metabolic overload of the competition phase and can complement conventional clinical indicators in the prevention/management of overtraining. In the same context, Jürimäe et al. examined selected myokine responses to an endurance rowing training session in national level female rowers. The study concludes that the acute negative energy balance, induced by a single endurance rowing training session, elicits significant increases in plasma irisin, fibroplast-growth-factor-21, and follistatin levels and suggests that these biological markers are useful for the assessment of acute exercise stress in female rowers.

The Concept 2 (C2) rowing ergometer is widely used for off-water training and performance assessment, and its popularity has grown even outside the sport of rowing, as it can be found in most health clubs. However, despite the wide use of the C2 there are relatively limited data on its validity and accuracy. The method of generating resistance in the C2, by air-dampening, implies that the targeted mechanical output is critically influenced by the rower’s effort and associated high variability (4–5% even in elite rowers, Treff et al., 2018) and, thus precludes any acceptable variability calculation due to lack of controlled rowing stroke parameters. However, Treff et al. controlled the inherent biological variability, by using a mechanical test rig (Mentz et al., 2020), and demonstrate that the accuracy of the C2 for a given mechanical power output is improved when the fluctuations in the rowing pace during the initial strokes of a rowing race are removed. Nevertheless, the significant underestimation of the first five strokes should be taken into account when conducting tests of short duration (e.g., 20s all-out effort) that are relevant not only when planning anaerobic training sessions, or as predictor of 2000 m rowing ergometer performances (Cerasola et al.), but also to the shortened race distance for the 2028 Olympic Games. The underestimation (∼10%) of the mean power output, as well as the first five strokes, compared to that performed by the rower, is confirmed by Holt et al. who investigated the concurrent validity and reliability of three commercially available on-water rowing instrumentation systems and the C2 in comparison to bespoke instrumentation.

Finally, Alfőldi et al. present anthropometric and physiological characteristics of Hungarian successful rowers and confirm the importance of body mass for rowing performance as previously shown (Secher and Vaage, 1983). It might be noteworthy that the reduction of racing distance planned for the 2028 Olympic Games would most likely exacerbate the influence of body dimensions on rowing performance and further make rowing a sport for tall rather than for all, with implications for the universality of the sport (Koshla 1983).

In conclusion, this Research Topic presents the resent developments in various aspects of rowing and elucidates enquiries concerning race pace, energy cost and requirements, immune responses, ergometer validity and performance limitations that can shape future training methodologies. Future enquiries should address the implication of potential metabolic limitation in training volume (Winkert et al.) and the suggested shift to higher proportion of high intensity training, especially with consideration for the danger of overtraining. Such considerations will become more relevant if the race distance is reduced to 1,500 m at the Olympic Games in Los Angeles in 2028 that will dramatically alter the metabolic profile of the sport. Additionally, aspects of CBF and oxygen metabolism during maximal rowing remain unresolved (Volianitis et al., 2020) due to technical limitations of evaluating methodologies. Future technological developments are expected to provide real-time measures of cerebral perfusion and metabolism during high-intensity and maximal rowing.
